# Cognitive Behavior Therapy at the Crossroads

**DOI:** 10.1007/s41811-021-00104-y

**Published:** 2021-02-08

**Authors:** Simon E. Blackwell, Thomas Heidenreich

**Affiliations:** 1 Mental Health Research and Treatment Center, Faculty of Psychology, Ruhr-Universität Bochum, Massenbergstraße 9-13, 44787 Bochum, Germany; 2 Esslingen University of Applied Sciences, Esslingen, Germany

**Keywords:** Cognitive behavior therapy, Third wave, CBT history, Psychological therapy

## Abstract

The early development of cognitive behavior therapy (CBT) can be characterized by the coming together of behavioral and cognitive traditions. However, the past decades have arguably seen more divergences than convergences within the field. The 9^th^ World Congress of Behavioural and Cognitive Therapies was held in Berlin in July 2019 with the congress theme “CBT at the Crossroads.” This title reflected in part the coming together of people from all over the world, but also the fact that recent developments raise important questions about the future of CBT, including whether we can in fact treat it as a unified field. In this paper, we briefly trace the history of CBT, then introduce a special issue featuring a series of articles exploring different aspects of the past, present, and future of CBT. Finally, we reflect on the possible routes ahead.

Since its initial inception to the present day, cognitive behavior therapy (CBT) has been characterized by an unending stream of developments in both its clinical practice and the scientific foundations on which this is based. These developments have led to a field that is now rather heterogeneous in nature, and one place where this increasing heterogeneity is particularly apparent is at conferences. A conference is where people share the very latest in research and clinical developments, and as such provides a valuable snapshot of the current state of a field. A conference also provides a space where clinicians and researchers from a huge diversity of backgrounds can mix and exchange their skills and knowledge, providing a fertile crucible for the generation of new ideas and new collaborations that will move the field forwards.

In July 2019, the 9^th^ World Congress of Cognitive and Behavioural Therapies (WCBCT 2019) was held in Berlin, Germany. With more than 4000 participants from over 80 different countries, it was the largest international CBT conference^1^ ever held, and included 18 scientific streams covering the major areas of research and clinical practice (see Heidenreich and Tata 2019; Heidenreich et al. 2019). The congress was given the title “CBT at the Crossroads,” in part to reflect the coming together and meeting of people from all over the world, and in part to reflect the many challenges—and decisions—facing CBT as a field. Putting together the scientific program in itself raised a number of observations that we, as Scientific Committee chairs alongside Philip Tata, found highly interesting. For example, in recent years, major research topics seem to have shifted—while anxiety disorders have played a major developmental role in the history of CBT, issues such as the treatment of post-traumatic stress disorder and emotional disturbance in children and adolescents seemed to have become more prevalent, as seen by the increasing numbers of submissions on these themes. We suspect that besides developments within the field (e.g., there may be comparatively little scope for improvement in already existing highly effective treatments for some anxiety disorders), these changes probably also reflect changes in the world, our societies, and therapeutic priorities. It struck us that the scale of the WCBCT 2019 congress, in terms of not only the breadth of ideas presented and discussed but also the act itself of bringing together in one place so many people from all over the world, was an invaluable opportunity to reflect on the state of CBT at the current time and take stock of where we stand at the crossroads—the routes traveled to get here; the convergences and divergences of paths; the interchange of ideas, insights, and skills; and the signposts indicating the many possible ways forwards. As the dust settled in the weeks and months after the congress ended, we thought about how we might be able to build on this opportunity. Our idea was to put together a special issue that highlighted some of the cross-cutting themes that arose from the symposia, open papers, and posters submitted to the congress, taking these as reflecting the current state of CBT. We planned that each article in the special issue would reflect an aspect of one of these themes, including how research or practice has developed in this area, the current state of the field, and future directions. Our hope was that this would thus stimulate discussion and provide inspiration for future work, as well as a valuable perspective on “CBT at the Crossroads” in 2019 to look back on in future years.

In this paper introducing the special issue, we will start with a brief outline of the history of CBT and the route it has taken to the current crossroads; from early applications of learning theory in the 1930s to the emergence of cognitive therapy in the 1970s and the convergence of these two traditions in cognitive behavioral approaches, there has always been a certain variety in approaches that can be hidden by the generic term “Cognitive Behavior Therapy.” We will then introduce the articles in this special issue and consider the various crossroads for CBT that they reflect. Finally, we will look to the future and the routes ahead for CBT, both as a therapy or set of therapies, and as a field more broadly.

## A Short History of CBT in Four Acts

As noted above, Behavioral and Cognitive Therapies are a family of approaches rather than a single one. This is reflected in the congress title of “World Congress of Behavioural *and* Cognitive Therapies” rather than “World Congress of Cognitive Behaviour Therapy.”^2^ While it is difficult to delineate the development of CBT in clear steps, there is a certain degree of agreement that important developments can be summarized in developmental phases (Rachman 2015, p. 1):

“The early attempts at behaviour therapy were followed by the development of cognitive therapy, and latterly by cognitive behaviour therapy (CBT). Behaviour therapy emerged in independent but parallel paths in the United States and the United Kingdom during the period from 1950 to 1970. The second stage, the development of cognitive therapy, took place in the U.S. from the mid-1960s onwards. The third stage, the merging of behaviour therapy and cognitive therapy into CBT gathered momentum in the late 1980s and is now well established in Britain, North America, Australia and parts of Europe.”

We will present these earlier developments in the form of classical drama.

### Act 1: Learning Theory, Behavior Modification, Behavior Therapy

The origins of modern CBT can be traced back to early applications of learning theory principles such as classical and operant conditioning to clinical problems. Stanley Rachman (2015, p. 1), in the paper in “Behaviour Research and Therapy” in 2015, nicely summarized these developments:

“The new approach, behaviour therapy (BT), was a major shift away from the prevailing psychiatric treatment for psychological disorders (mainly medications and physical treatments), and fundamentally different from the psychoanalytic method.”

As is characteristic of new scientific developments, new journals were founded (“Behaviour Research and Therapy” in 1963) and the acronym BRAT perhaps reflects what other established forms of therapy thought of this “new kid on the block.” The earliest accounts that applied learning theory principles reach back to the 1920s in the famous account of conditioning emotional reactions in “Little Albert” by Watson and Rayner (1920). Behavior Therapy continued to develop during the following decades (e.g., with Wolpe’s 1958 book on systematic desensitization) and became established with the first journal, BRAT, as mentioned above, and formation of the first national and international associations (e.g., VGT and AABT in 1966, EABT in 1971). With growing evidence of its effectiveness and efficacy, one might have predicted that this form of Behavior Therapy might prevail. Yet, as is often the case in history, new trends emerged at the side of the stage…

### Act 2: Cognitive Therapy

In the USA, Albert Ellis and Aaron T. Beck were both trained in psychodynamic psychotherapy but became dissatisfied with basic assumptions of this approach and started new developments: Aaron T. Beck started what would later be known as “Cogntive Therapy” and Albert Ellis (1962) founded “Rational Emotive Therapy.” Where Behavior Therapy stressed conditioned reflexes and the control of behavior, cognitive therapy stressed the importance of internal events such as (dysfunctional) thoughts, belief systems, conditional, and unconditional assumptions. Beck (1970, p. 185), in a paper in “Behavior Therapy,” summarized these new developments and their relation to Behavior Therapy:

“Despite the fact that behavior therapy is based primarily on learning theory whereas cognitive therapy is rooted more in cognitive theory, the two systems of psychotherapy have much in common.”

Beck (1970, p. 186) after discussing a number of similarities between BT and CT, nevertheless remarks on differences on a theoretical level:

“The most striking theoretical difference between cognitive and behavior therapy lies in the concepts used to explain the dissolution of maladaptive responses through therapy. Wolpe, for example, utilizes behavioral or neurophysiological explanations such as counterconditioning or reciprocal inhibition; the cognitivists postulate the modification of conceptual systems, i.e., changes in attitudes or modes of thinking.”

As we will see, these differences did not stop the two approaches from moving closer together. Still, it must be noted that not all Behavior Therapists adopted the emerging new paradigm of CBT: What would later emerge as “Acceptance and Commitment Therapy” (Hayes et al. 1999) stuck closer to the Behavioral roots by refining Skinner’s functional approach (e.g., Skinner 1945).

### Act 3: Cognitive Behavior Therapy

There is little need to explain what can be thought of as “modern CBT” today as it is the dominating form of behavioral and cognitive therapies at this point in time. Rachman (2015) cites David Clark’s cognitive theory of panic disorder as the first instance in which these two traditions were brought together (Clark 1986). Rachman (2015, p. 6), again, nicely summarizes this confluence and how it changed both streams:

“In the process of merging BT and cognitive therapy, the behavioural emphasis on empiricism was absorbed into cognitive therapy. The behavioural style of conducting empirical outcome research was adopted, with its demands for rigorous controls, statistical designs, treatment integrity and credibility, and so forth. In turn, cognitive concepts were absorbed into BT, and cognitive therapists made greater use of behavioural experiments.”

By the end of the 1980s, CBT was firmly established with increasingly methodologically stronger trials showing the effectiveness and efficacy of this treatment approach for a variety of mental disorders and physical problems. In the terminology of Thomas Kuhn (1962), this work looked like it could provide a potentially overarching paradigm that could guide CBT research (and dissemination) over the following decades. The decision to combine the World Congress on Behavior Therapy and the World Congress of Cognitive Therapy for the first WCBCT in Denmark in 1995 further reflected a clear statement of intent on the part of clinicians and researchers from the behavioral and cognitive traditions to share a road forward in the continued development of CBT.

However, new developments that would introduce previously unheard of concepts and methods into the behavioral and cognitive therapy family were again preparing for their appearance…

### Act 4: Developments Since the Early 1990s: Schema Therapy, ACT, Mindfulness

At the same time as CBT was becoming established in an increasing number of countries and health systems across the world, eminent researchers and clinicians again realized that the original assumptions of CBT might not fit the needs of all clients: In this vein, Jeffrey Young, a former colleague of A.T. Beck, published his book “Cognitive therapy for personality disorders: A schema-focused approach” in 1990 (Young 1990). Although at the time he still called it “cognitive therapy,” this approach would soon develop into schema therapy. Young argued that standard CBT makes a number of basic assumptions (e.g., that the patient is able to form a conventional therapeutic relationship based on the cognitive model) that often do not work well with patients diagnosed with personality disorders. Young introduced methods from other schools of psychotherapy (e.g., interpersonal approaches) into his new approach. Around the same time, Marsha Linehan, also working with young patients with diagnoses of personality disorder, recognized enormous challenges in the treatment of borderline personality. In her approach (dialectic behavior therapy, DBT; Linehan 1993), she stresses the necessity of balancing behavior change principles (such as contingency management) with acceptance skills (such as mindfulness). Acceptance and mindfulness have also played a role in what in 1999 would be published as “Acceptance and Commitment Therapy” (ACT). Developed by Steve Hayes and his group in Reno, Nevada, this approach did not believe in the “C” in “CBT” but rather kept working in the functional contextual approach introduced by Skinner. To name one final example of these developments, Zindel Segal et al. (2002) developed mindfulness-based cognitive therapy (MBCT) for the prevention of depressive relapse.

These new developments were seen as highly promising by some colleagues, while others feared that the CBT therapy field would become overly heterogenous. Steve Hayes coined the term “third wave” to characerize this new direction in cognitive and behavioral therapies:

“The third wave reformulates and synthesizes previous generations of behavioral and cognitive therapy and carries them forward into questions, issues, and domains previously addressed primarily by other traditions, in hopes of improving both understanding and outcomes” (Hayes 2004, p. 658)

We now conclude the historical section of this paper and will move on. There is much more that could be said about the development of CBT, including many more storylines, subplots, and both leading and supporting actors. In fact, such a broad-brush retelling invariably focuses on a few key figures, whereas there are of course many other clinicians and researchers who made crucial contributions to the development of the ideas and techniques that make up what we currently think of as CBT. Some of these contributions are well-documented in the literature, while others may be fondly remembered by colleagues, supervisees, and students, but otherwise assimilated, unattributed, into the broader knowledge base.

Alongside the largely conceptual developments in relation to cognitive and behavioral therapies that we have outlined above, there have been many other kinds of developments, for example, in how CBT or elements of CBT have been disseminated as interventions. A striking example of this is via the use of technology: From face-to-face CBT to telephone, live chat, or video-call based therapy, or from self-help (e.g., books) through to computer-based, internet-delivered or app-based self-help interventions based on CBT (e.g., see Ström et al. 2000, for a pioneering example). Other developments include the spread of CBT-based interventions to a broader range of problem areas and populations, but with more specific tailoring.

This development of cognitive and behavioral therapies over nearly a century (if we are willing to count Little Albert as an early manifestation of BT) with the merging of the behavioral and cognitive traditions into CBT, followed by yet new developments and the major impact of new technologies, inspired the Scientific Committee Chairs of WCBCT 2019 to choose “CBT at the Crossroads” as the congress theme.

## Introducing the Special Issue Contributions: Perspectives from the Crossroads

To put together this special issue, we drew on our knowledge of the WCBCT 2019 scientific program, and consulted with colleagues in the Scientific Committee, to identify a range of potential key themes or areas of interest arising from the conference content, and for each we invited someone to contribute an article. We invited people who made a contribution to the congress that broadly fell within the chosen theme, and thus we knew would have expertise and a perspective to impart. In addition to contributions from well-established senior researchers and clinicians, we also sought contributions from people at relatively early or “mid” stages of their careers, including those who did not yet have a long history publishing in their respective area, in order to gain a combination of both experience-rich and fresh and future-oriented perspectives.

As you will soon see, some of the contributions address very broad themes, and others focus on a very specific topic that provides an example of a broader subject area. The articles in the special issue of course reflect only a small sample of the richness of ideas and interchanges represented at WCBCT 2019. For example, we deliberately took a broad-brush rather than disorder-specific perspective, but within many specific disorder and problem areas there have been a huge range of exciting developments with great future potential. Similarly, there are many types of therapy within the broader CBT family, such as schema therapy, ACT, and mindfulness-based CBT interventions, which have seen great advances and are highly relevant for the future of CBT. There are also specific populations, such as older adults, or areas relevant to CBT, such as training and supervision, not represented in this special issue. Most probably every individual reader could come up with something they saw as a hot topic at the conference that seems to be obviously missing! We hope that our choice of themes is sufficient to stimulate thought and discussion about the broad and diverse family of CBT approaches—and of course some discussion may in fact be provoked by considering what is *not* present—but it is not intended to be an indication of which areas we think are more or less important or pressing.

The following section introduces the special issue papers, placing them at one or other crossroads for CBT, and reflecting on the broader associated themes, challenges and opportunities for CBT they represent.

### The Interface of Technology and Therapy

One trend in CBT that has accelerated since perhaps the 90s onwards is how technological advances have been used for therapy purposes—from computerized interventions distributed on floppy disks or CD-ROMs (see e.g., Marks et al. 1998) through to internet- and smartphone app–based treatments (see e.g., Andersson et al. 2019; Linardon et al. 2019). When we think of CBT or psychological therapy more broadly, most of us will by default imagine a face-to-face interaction between patient and therapist, but as years of research now show, there are plenty more ways in which the principles of CBT can be made use of to effect therapeutic change. The meeting of CBT expertise and technological advancements is therefore one that has led to dramatic transformations in how we do therapy, and will continue to do so.

Lindner (2020) provides an overview of one such example of an interface between technological advances and CBT, the use of virtual reality (VR) in therapy. As Lindner notes, this is an area where the developments in the technology itself have had a major impact on what is possible and also the potential for dissemination. What until quite recently seemed very much future technology is becoming more and more a potentially feasible option for treatment delivery. As the technology will undoubtedly continue to develop at a rapid pace, so will the possibilities offered for CBT.

Also building on technological developments is the approach reviewed by Rasing (2020), blended CBT, here reviewed specifically in relation to adolescent depression. In blended CBT, face-to-face sessions are supplemented by internet- or smartphone-delivered therapy components outside of the sessions. Given that the use of such technology is a standard part of the everyday lives of most adolescents (and also adults), to some extent it would seem a missed opportunity not to make use of this within therapy. However, questions about how best to do this remain to be explored.

Although technological developments present great opportunities for CBT, they also come with challenges. One challenge concerns the potential risks that may accompany any new development. In recent years, there has been increasing awareness within psychotherapy research more broadly of the need to consider negative effects and harms (e.g., Parry et al. 2016), and it may be that there are specific risks, negative effects, or contraindications associated with newer technology-delivered versions of CBT. The need to collect data on potential negative effects and risk factors associated with use of these newer technologies has been recognized within the literature, for example, in relation to CBT delivered via the internet (e.g., Rozental et al. 2014) or assisted by virtual reality (e.g., Rus-Calafell et al. 2018). This is important not only to avoid unnecessary harm but also to avoid people being unnecessarily denied the opportunity to benefit from new modes of treatment due to untested assumptions and fears about their suitability or safety for certain groups of individuals.

A second challenge relates to the knowledge and skills required by a clinician or researcher when conducting therapy or developing new interventions. How much knowledge or skills in the new technology, or at least its use, will be needed by individual clinicians or researchers in addition to their expertise in the “traditional” aspects of the therapy itself? And how can individual clinicians and researchers keep pace with rapid technological developments on top of other ongoing training needs? It seems likely that the greater the use of technology in therapy, the more clinical services and research teams will need to count specialist technicians, computer scientists, and software developers amongst their members. This in turn can bring increased opportunities for interdisciplinary interchange and introduction of new ideas into CBT development.

A third challenge from technological development is not so much about how to use technology in CBT, but rather its influence outside of the therapy context; the more ubiquitous technology is, and the greater the role it plays in individuals’ everyday lives—whether via computers, tablets, phones, watches, or other devices—the more therapy (and the theories informing it) needs to be adapted to take these into account. For example, if someone’s social life and identity is inextricably tied in with use of technology (e.g., via social media or messaging services), this needs to be considered in assessment, formulation, and treatment. In turn, the ubiquity of technology such as smartphones in daily life offers many possibilities for convenient and ecologically valid collection of data on people’s day-to-day or moment-to-moment experiences to inform therapy. As technology develops, so too will the diversity of ways in which it plays a role across the lives of different individuals; this provides challenges for CBT in keeping pace, but also many valuable opportunities to help people make changes where it really counts—outside of the therapy sessions.

### From Treatments for Disorders to Treatments for Individuals

One of the crossroads at which clinicians will find themselves almost every day in their practice is that where an evidence-based treatment protocol or set of techniques meets the complex presentation of an individual patient. We will all be familiar with the questions of “what works for whom,” and for any given diagnosis most treatment guidelines can provide a list of potentially efficacious interventions. However, matching a diagnosis (e.g., “major depressive disorder”) to a treatment (e.g., “CBT”) has obvious limitations: We know that there is large heterogeneity within diagnostic categories, that a significant proportion of patients receiving any particular form of CBT will not respond, and also that “CBT for depression” (or another disorder) can cover a wide range of CBT-based treatment approaches. A number of papers in this special issue address some aspect of this dilemma of trying to match treatments more precisely to individual patients.

Lorenzo-Luaces et al. (2020) tackle an issue that was much discussed at the conference and has been an increasingly hot topic in the past decade or so—personalization of treatments for depression. Given the notoriously heterogeneous nature of depression, matching patients to very different treatments, such as CBT vs. SSRIs, seems like something that is not only necessary, but should be very possible. However, as noted by Lorenzo-Luaces et al. (2020), this endeavor has faced, and continues to face, many challenges. Although the focus is on depression, this paper provides a broader exemplar of how both clinical and research thinking has had to adapt and change as the initial assumptions have not always met with the success anticipated. This mirrors broader discussions within medical literature about how statistical-driven approaches to treatment personalization have so far often not lived up to expectations (e.g., Wilkinson et al. 2020). Based on their review, Lorenzo-Luaces et al. 2020) make a number of suggestions for moving forwards, including new methods for the assessment of potentially predictive patient characteristics, and consideration of what kinds of treatment comparisons may provide the greatest opportunities for detecting differential patient-treatment outcomes.

Another facet of matching treatments to individuals concerns the role of diagnostic categories. The past 40 years or so have been dominated by treatment protocols developed with specific diagnostic categories in mind: CBT for depression, CBT for social anxiety disorder, CBT for PTSD, and so on. While treatments focused on individual disorders have shown to be highly effective for the reduction of symptoms, high rates of co-morbidity remain a challenge. In the research world, studies have tried to understand psychopathology by comparing the characteristics of patients with one diagnosis to those with another. However, particularly in the past 20 years there has been a marked increase in attempts to understand mechanisms, and develop treatments, that cut across different disorder categories, i.e., that are transdiagnostic. In this special issue, Schaeuffele et al. (2020) address the concept and development of transdiagnostic treatments, which they categorize broadly as “one size fits all” vs. “my size fits me” approaches. From the perspective of the transdiagostic approaches reviewed, the question of which treatment for which individual is not something that always needs to be asked at the level of diagnosis, but rather leads to broader questions as to whether one generic approach can be developed that in fact can be applied successfully to a broad range of problems, or whether modularized approaches, made up of several specific targeted treatment components, can achieve a sufficient level of individualization to be successfully matched to a wide range of individuals with diverse problems.

Touched upon within the paper by Schaeuffele et al. (2020) are closely related questions that are not so much about diagnosis-driven versus transdiagnostic approaches, but rather about self-contained therapy “packages” versus principles. CBT treatments are based on a set of underlying theoretical principles and ideas about mechanisms and how to target them in therapy. Early approaches (e.g., behavior therapy) generally designed a treatment program for an individual from these first principles (e.g., via a functional analysis), and this approach of course continues to be taught and applied. However, over time CBT principles and techniques have generally been collected together and bundled into specific treatment packages, sometimes even acquiring a specific “brand name.” The creation of such treatment packages is in itself not necessarily a bad thing and helps provide useful heuristics and short-cuts for providing efficient and targeted treatments. There are also many contexts (e.g., simple presentations of specific phobias) where a narrow focus is often desirable and it is over-complication that needs to be avoided. However, recently there have been renewed calls to try to move away from “brand name” therapies and specific techniques for particular disorders, and instead organize our approach to conceptualization and intervention at the level of specific processes or mechanisms (e.g., Hofmann and Hayes 2019; Holmes et al. 2018). Many will argue that this is what they have been doing all along, but this has not been reflected in the broader field: To date, an ultra-flexible individualized CBT approach superior to disorder-specific treatment manuals has not yet materialized, or at least has not yet entered the mainstream as a recommended frontline treatment. Challenges include providing a new template for treatments while avoiding simply becoming one more competing “brand,” and moving from broad overarching treatment principles to something concrete that can be trained, tested for efficacy, and successfully disseminated. However, the amount of work and energy currently being invested in this area holds promise for future success.

Another important aspect of tailoring of treatments to individuals that can sometimes be neglected is reflected in the adaptation of CBT approaches to groups of people with different needs. This is illustrated in the special issue by the paper by Hronis (2020), which reviews the state of CBT as adapted for people with intellectual disabilities. As she notes, this is an area with a relative lack of research, but a number of new approaches are arising that build on some of the developments seen within CBT more broadly, including third-wave approaches and development of computerized interventions specifically tailored to children with intellectual disabilities, who represent a population with large unmet treatment needs. Therefore, as CBT approaches develop and hopefully improve—including better tailoring of treatments to individuals or specific mechanisms of interest—it is important that these advances also benefit these more vulnerable groups of individuals who are often not the first-line recipients of mainstream research attention.

### Basic Research and Clinical Practice

If we wish to target specific processes and mechanisms in our therapies—as argued for by a mechanisms- or process-focused approach as mentioned above—then the questions arise of how we can identify, detect, or assess those that are relevant, and how we can target them precisely and efficiently. It is often hoped that answers to these questions may be found at the crossroads of basic research and clinical practice, and this special issue contains several articles exploring these possibilities.

Ouimet et al. (2020) address one strand of research for which mechanisms are perhaps the key focus—experimental psychopathology. They provide examples of how experimental research has provided insights into mechanisms of both disorders and therapy, helping to improve the effectiveness of CBT approaches. However, they also note challenges, cautionary tales, and potential pitfalls for this area of work. Looking to the future, their recommendations include two that are clearly highly relevant not just in experimental psychopathology but across the whole of CBT more broadly. One of these concerns the importance of robust research methods and a commitment to open science. The other concerns the need to include more diversity in our research samples if we wish to develop models and treatments that can generalize beyond the restricted range of people who traditionally take part in research studies.

Blackwell (2021) highlights one specific area—mental imagery—and examines the various ways in which mental imagery has been explored at a number of different interfaces between science and clinical practice in CBT. These range from the use of imagery in experimental studies to elucidate mechanisms of psychopathology, to the development of therapeutic techniques such as imagery rescripting. While mental imagery–based techniques have been used for a long time across many different psychological therapy approaches, in recent years there seems to have been a particular drive to dissect their mechanisms and develop more tightly targeted techniques for use within CBT.

The paper by Månsson et al. (2020) also examines on one particular intersection between science and practice—the use of neuroscience, including neuro-imaging techniques, to inform, develop, and tailor CBT approaches. This is an area that holds great potential, and has generated much excitement, but not always lived up to the earlier promises; Månsson et al. (2020) review some of the ways in which neuroscience may help improve CBT outcomes, and make suggestions to increase the utility of this work in future. Some of the examples they choose for illustration are not without controversy, and will likely be met with divided opinions as to their level of success or future potential. Nevertheless, Månsson and colleagues provide useful exemplars for some of the many different ways in which neuroscience may inform CBT development, and hopefully lead to better outcomes in future. Similar to Ouimet et al. (2020), Månsson et al. (2020) also highlight methodological improvements, including steps to increase reproducibility, as crucial for the future success of the field.

While these three articles make important points about the need to understand mechanisms in CBT, and how basic and translational science can play a key role, they also illustrate one important aspect of a conference such as WCBCT: the representation of work across the whole spectrum from basic and experimental research to clinical trials, skills classes, and workshops. As has been noted, it is all too often the case within the broader field of mental health that people in different disciplines tend to go to different conferences, encountering only a narrow range of work, and this contributes to a siloing of interests and expertise (Holmes et al. 2014). While, for example, research on neural representations or experimental manipulations of cognitive processes may not have immediate relevance for what a clinician does with the patients they see the following week, it is only by bringing basic research to clinicians, and clinical experience and expertise to basic researchers, that research can inform clinical practice, and vice versa. By providing the opportunity for cross-talk and cross-fertilization of ideas and expertise between researchers and clinicians, CBT conferences such as WCBCT play a crucial role in advancing both the science and practice of CBT.

### Beyond a Symptom Reduction Focus to Broader Aspects of Existence and Well-being

During the last few decades, cognitive and behavioral therapies have often focused on symptom reduction as the main target for therapy. While this is arguably the major reason for therapy and for health insurance to pay for these treatments, this narrow focus may well run into problems: in a number of disorders such as recurrent depression and addictions, it has been noted that long-term improvements (and the maintenance of therapeutic gains) usually require broader areas of the patient’s lives to be well balanced. While third-wave approaches such as ACT (Hayes et al. 1999) have emphasized the realization of individual values, this broader perspective is not yet consistently represented in the CBT mainstream. Opening up our perspective to broader aspects of patients’ lives brings issues to our attention that have so far been mostly part of other therapy traditions.

During the last two decades or so, Giovanni Fava and his group in Bologna have developed a relapse prevention treatment for recurrent depression, arguably the most significant mental disorder across the globe. While CBT and other psychotherapy approaches such as interpersonal therapy have been shown to be highly effective for the treatment of depressive episodes, it is much harder to help patients stay non-depressed in the months and years following this treatment. In this special issue, Guidi and Fava (2021) describe well-being therapy (WBT), especially developed for patients with recurrent depression. Rather than focusing on reducing distress via behavioral activation, cognitive restructuring etc. (therapy techniques prominent in the treatment of acute depressive episodes), the emphasis is on enhancing patient’s well-being, defined using the model of Ryff (1989), which stresses factors such as self-acceptance, personal growth and purpose in life, autonomy, interpersonal relationships, and environmental mastery. Guidi and Fava (2021) outline the development and current state of this approach. In highlighting some of WBT’s particular characteristics, which include not only the emphasis on well-being itself but also other factors such as a “staging” approach to treatment planning, Guidi and Fava raise a number of important concerns that have relevance to the wider field of CBT, beyond WBT itself.

Opening the perspective to broader aspects of human lives naturally brings topics to our attention that have long been part of the traditions of other therapeutic approaches. One such approach is existential psychotherapy and existential phenomenology. While the cognitive and behavioral traditions and existential approaches were for decades highly critical of each other (mostly due to different philosophies of science and basic assumptions), the last few years have seen a rising interest in existential issues from a number of CBT colleagues across the globe. In a paper based on a panel discussion at WCBCT 2019, Heidenreich et al. (2021) explore the potential of existential approaches for cognitive and behavioral treatments and therapy. They relate the history of both approaches and the few encounters over the decades and then go on to discuss implications for our understanding of psychopathology (e.g., the potential role of death anxiety for anxiety disorders and OCD) and implications for training and supervision.

### A Global Perspective on CBT

The global spread of CBT has increased rapidly over the past few decades, and this has resulted in a number of particularly interesting crossroads reflecting the meeting of different cultures and customs. Correspondingly, the cross-cultural adaptation of CBT was, unsurprisingly, very strongly represented at WCBCT 2019. In this special issue, we have two exemplars of such adaptation, both from Latin America. The first paper by Neufeld et al. (2021b) describes the development of CBT over Latin America more broadly, and the second by Neufeld et al. (2021a) hones in specifically on Brazil as a more detailed exemplar. These papers illustrate a number of the challenges in introducing CBT to a new country—or set of countries—and some ways in which these challenges can be overcome. While the papers are primarily concerned with the adaptation of CBT to a particular country, they also make the important point that in fact cultural adaptation of CBT is not only something that may have to happen between countries but also within countries and communities, as no country itself represents one homogeneous culture or population. The WCBCT program included not only work on cross-cultural adaption of CBT to other countries—with Asia and Africa also strongly represented—but also adaptations to particular cultural, ethnic, or religious groups within specific countries, and to other aspects of diversity including sexuality and gender that are central to individuals’ identities. Alongside adaptations to the therapy itself it is also crucial to take steps to increase diversity amongst the CBT workforce, from trainee therapists through to service leaders; this is one area where CBT organizations and training institutes have an important responsibility. Such work is indispensable to making CBT a universally relevant treatment, and is in fact a central concern of the next World Congress (theme: “East Meets West: Embracing diversity and improving access to CBT”), alongside global dissemination, use of new technology, and other innovations. It is very encouraging for the future of CBT that themes of diversity are the focus of so much energy.

While the global spread of CBT may often be portrayed in terms of CBT “exporters” and “importers,” it is by no means a one-way street, or at least it should not be if we want to make the most of the opportunities available. For example, the inventiveness and resourcefulness required to disseminate CBT in places where there is a lack of trained mental health professionals has led to approaches involving delivery via lay health workers with a relatively small amount of training (e.g., Gureje et al. 2019), including in non-standard settings such as the “friendship bench” approach (Chibanda et al. 2016); these and related approaches could readily have applications to increase accessibility of CBT-based interventions in countries where standard CBT is well established in health services (e.g., Elbert et al. 2017). Furthermore, as CBT continues to be developed and research studies or programs established in countries where CBT is still relatively new, there will undoubtedly be many innovations and ideas arising that can inform improvement of CBT in countries traditionally seen as “exporters.”

One challenge posed in trying to adapt CBT treatments from one country to another is posed by the vast differences in health systems and services, and the ways in which psychological therapies may be accessed or paid for—for example, whether via state funding, health insurance, or only privately. There are also related differences in who exactly is authorized to provide CBT, how many sessions may be available, and the freedom or restriction placed on how exactly the therapist is required to deliver the therapy. Ideally, provision of CBT across the globe would follow the accumulation of evidence for its effectiveness and cost-effectiveness. However, in reality, the situation is far more complex and often muddied by a web of financial, political, and protectionist professional interests. This is where CBT and other organizations can come into play, whether national, trans-national, or indeed global, such as the World Confederation of Cognitive and Behavioural Therapies (WCCBT; https://wccbt.org/), formally launched at WCBCT 2019. These organizations have an important role, not only in disseminating CBT evidence and training but also through coordinated sharing of experience and expertise in the organizational and political aspects of making CBT available. The danger here of course may be if CBT organizations become yet another self-interested lobby group whose primary purpose is self-promotion. Taking a critical perspective and remaining committed to following the evidence is therefore essential.

## CBT at the Crossroads: Navigating the Future Paths

If the contributions to this special issue, as discussed in the previous section, represent at least some aspects of the crossroads at which CBT currently finds itself, where do we go from here? In this final section, we will consider some broader questions for CBT as we go forward and leave the WCBCT 2019 conference further behind us.

### What Do We Mean by CBT?

As noted earlier, although we often talk of CBT, in reality, it is more accurate to speak of Cognitive and Behavior Therapies—as reflected in the name of the congress. The articles in this special issue and the program of WCBCT 2019 illustrate how the term CBT can encompass a range of disorder-specific or transdiagnostic protocols, techniques- or mechanisms-focused approaches, and therapies on a continuum emphasizing more or less behavioral to cognitive techniques, as well as schema therapy, ACT, mindfulness-based interventions and more. Does it then still make sense to speak of “CBT” as if it is a unified field, or are there irreconcilable differences that mean we need to reconsider what exactly we are terming CBT?

It is clear that there is no one single therapy protocol that one can claim is “the” CBT, and from this perspective defining CBT by what it involves *doing* is bound to run into unresolvable arguments and unhelpful hair-splitting. In fact, it may be most productive to consider defining CBT in terms of principles rather than specific procedures—for example, that it is based in a scientific understanding of cognitions, behavior, emotions, physiology, and their inter-relationships with the broader social environments in which people live; that it aims to effect change in the present (or near future); and that it remains committed to generating, and being grounded in, empirical data. There is probably much more that could be added, for example, values inherent to CBT such as the intrinsic worth of every individual, and the importance of empowering individuals to make the decisions and changes (or lack of them) that they wish to make in their lives.

Whichever elements may fit into such a definition, and how exactly they are best formulated, defining CBT in this broad way, based on principles rather than simply procedures, not only allows inclusion of the broad family of approaches as represented at the WCBCT 2019 conference but allows CBT to avoid becoming stuck in the past, instead freeing it to continuously adapt and develop alongside the science and evidence base. If a therapeutic technique is developed outside of the CBT field that appears highly effective, CBT researchers and clinicians should not simply dismiss it as “not CBT”, although neither should they take the “eclectic” approach of simply incorporating it wholesale. Rather, they should work on how to include it within a CBT framework—if necessary, adjusting this explanatory framework to fit—and then testing whether its inclusion does indeed improve outcomes (see e.g., Clark 2010). One such example is the “Cognitive Behavioral Analysis System of Psychotherapy” (McCullough Jr. 2003), which incorporated and adapted psychodynamic transference principles into a CBT framework. One consequence of taking a broad principles-based definition of CBT is that CBT in 100 years’ time may look very different from the CBT of today, but still be identifiable as CBT; in fact, we should hope that this will be the case, otherwise we will have made very little progress.

While the proliferation and continued development of more and more CBTs may present a definitional challenge, it in fact reflects a positive aspect of CBT, in that it is characterized by continuous development, creativity, and a drive to achieve ever-better outcomes. It also hopefully reflects a relatively open and democratic research environment in which anyone can contribute to and build upon the evidence base. From the perspective of conferences such as WCBCT, to have a diversity of views and approaches coming together in one place can only be a good thing in terms of the potential for exchange of ideas and challenging of pre-conceptions or assumptions. However, with the increasing success of CBT and its subsequent popularity and expansion across services, countries, areas, and modes of delivery, there is also an increasing challenge in interpreting and using the evidence base: We may make statements like “CBT is effective for [x disorder],” and perhaps point to a meta-analysis including dozens of studies to back up our point. But here we come across the problem of what exactly we mean by “CBT”—which CBT or CBTs were included here? And is it the same CBT as the CBT we are offering or advocating? As CBT as a field grows, so too does the need for precision about how we speak about our interventions and the statements we make about them—not all CBT approaches will be equally effective. The diversity of approaches that can be grouped under the term “CBT” is undoubtedly a strength; the challenge is working out how we can ensure that the greatest number of individuals receive the best possible CBT for them.

### The Past, Present, and Future of the Evidence for CBT

The success of CBT also leads to a further challenge for treatment development and the generation of evidence. When treatment development in CBT (or Behavior Therapy) first started, there was often very little in the way of effective treatments for many of the disorders studied. Nowadays, for most disorders or problem areas we have treatments—sometimes a multitude of treatments—that are moderately effective. New treatments therefore now have much greater hurdles to overcome: First, they must provide justification for their existence in the context of the already crowded fields of evidence-based treatments, and second they need to find a way to make the transition from being evidence-based to being actually implemented in services (see e.g., Dunn et al. 2019). As Pim Cuijpers has expressed in the context of depression (Cuijpers 2017), if our goal is to reduce the burden of mental disorders, then it is highly unlikely that the best way to achieve this is via developing yet more new treatments. In fact, where we have very effective treatments there are other, most probably more fruitful, routes for increasing their impact on disease burden, for example, via optimizing dissemination, training, supervision, and increasing accessibility. However, given that there is still much room for improvement in our outcomes, what is the best route for continued treatment development and optimization?

This is of course not a straightforward question to answer, but it is one that researchers have been addressing from a number of different angles in recent years. Some potentially promising ideas in evidence at WCBCT 2019 included the ever-growing interest in network analysis approaches to the conceptualization, measurement, and modeling of underlying processes or symptoms in psychopathology (e.g., Bringmann et al. 2013), and a resurgence of interest in idiographic and single case-level approaches (e.g., Fisher and Bosley 2020; Kaiser and Laireiter 2018). Other developments include using a broader range of trial designs to answer different questions around mechanisms and optimization of multi-component interventions (e.g., Watkins et al. 2016), adaptive designs that allow addition and replacement of arms in an ongoing trial (e.g., Blackwell et al. 2019), and designs intended to test sequential allocation to different treatments (e.g., Nelson et al. 2018). While we do not yet know the extent to which any of these ideas will bear fruit in terms of facilitating treatment development and optimization, it is clear that in addition to the important work of developing new treatments, there also needs to be ongoing work as to how best we carry out this treatment development process.

Alongside the challenge of generating evidence for new approaches is the challenge of what to do with the evidence for treatments we already have. As the evidence base grows, the studies conducted recede into history and we may need to start asking whether they can still inform our views of the therapies we conduct today: To what extent can we expect trials from the 70s, 80s, or even 90s to be informative to our therapy choices now in 2020 and beyond, when the world and potential social and environmental determinants of mental health outcomes may have changed so much? One possible way to surmount this challenge is the continued collection of “live” evidence. An exemplar comes from the Improving Access to Psychological Therapies (IAPT) service in the UK, in that the collection of standardized outcome data is embedded into the service provision and examination of this has been used to inform improving outcomes (e.g., Clark et al. 2018). Another example comes from the setting up of the “KODAP” initiative to coordinate and standardize outcome data across university outpatient psychotherapy centers in Germany (e.g., Velten et al. 2017), and use of routine data collection to investigate innovations in treatment selection (Lutz et al. 2020). While discussions of “Evidence Based Practice” and “Practice Based Evidence” are of course nothing new, some of these more recent initiatives provide opportunities to combine these in increasingly innovative ways at a large scale that should hopefully enable continued updating and optimization of the provision of CBT, particularly if randomized designs can be incorporated to allow evaluations of any improvements made.

### Perspectives on CBT from a Pandemic

Although it was not on our agenda when we first started planning this special issue, it is now impossible to reflect on the future of CBT without considering the impact of the COVID-19 pandemic. The huge loss and disruption to lives that have resulted both directly and indirectly from the pandemic have had a number of impacts of relevance to the future of CBT. An obvious one of these has been the necessity to adapt to restrictions on movement and interpersonal contact, for example, entire face-to-face therapy services converting to delivering therapy and supervision via video conferencing software with a rapidness that would have been previously unimaginable, as well as workshops and whole conference programs going entirely online often for the first time (such as the 50^th^ Anniversary Congress of EABCT, which was held virtually rather than, as planned, in Athens, see https://eabct2020.org). Even when the pandemic ends, it is difficult to imagine that CBT (or much else) will simply return “back to normal”—rather it is likely that services, training, and conference organizations will capitalize on the new resources and ways of working that have been developed, and we will see much more in the way of hybrid and blended offerings. A positive side effect of this may therefore be a massive increase in the future accessibility of CBT (see e.g., Clark 2020). Furthermore, in relation to conferences, although many of us feel that nothing can truly replace the opportunity to get together with researchers and clinicians from all over the world in person, this is of course not an option for many people. The possibility of combined in situ and live-streamed conferences will undoubtedly open up these events to a much broader and more diverse group of people, and this is to be welcomed.

A second more indirect impact of the pandemic has been to show the huge value of CBT, both as a therapy, and in terms of the broader principles and characteristics of the field. For example, we have evidence-based interventions that can be adapted to the demands and needs of the pandemic, such as in formulating or treating the traumatic experiences of emergency workers or the complex grief of the bereaved, and we have often well-tested models and frameworks that can be used to understand and provide interventions and public health messages to reduce distress and impairment in relation to COVID-19. The pandemic has also brought to the fore the field of behavioral medicine and the importance of CBT to health and illness more broadly, for example, in understanding the ways in which adaptive anxiety around illness can tip over into being extremely distressing and impairing (e.g., Haig-Ferguson et al. 2020). Furthermore, given the potential longer term consequences of COVID-19 infection (e.g., Yelin et al. 2020), applications of CBT within the context of long-term conditions are likely to become even more relevant.

The responses so far to the pandemic have also illustrated some of the other great strengths of the field of CBT: rapid and versatile adaption of evidence-based approaches to new problems (e.g., Cambridge University Press 2020); immediate attempts to collect data and use science as the basis for further decision-making; bringing the science informing CBT to inform advocacy, political advice or lobbying, and public communication of science; and in many cases leading CBT centers generating and making publically available a host of resources such as treatment manuals, videos, and webinars for rapid dissemination (e.g., Beck Institute 2020; OxCADAT 2020; World Confederation of Cognitive and Behavioural Therapies 2020). Of course, out of the numerous valuable resources that were made available in a very short time-period, we can only mention a few, so these represent a small selection of English language materials. However, they hopefully illustrate our general point. Such a readiness to react and respond, combined with a commitment to science and a willingness to give away expertise are, we would hope, hallmarks of CBT as a field, and stand us in good stead for the future.

### CBT: “Just” a Therapy?

As emphasized via the congress theme, CBT can be thought of as at the crossroads not only in relation to development and dissemination of therapy but also with regard to pressing global issues, including ecological and political challenges, which we would argue CBT leaders and associations need to address, both in terms of promoting a better understanding and in identifying solutions for the effects of these challenges. When we talk about CBT we are of course primarily talking about psychological therapies—something that is done with individuals, families, groups, or organizations to bring about change. However, working in the field of CBT means more than just doing or researching this therapy. There are of course many problems for which the solution is not CBT, and promoting CBT is not about suggesting that CBT is always the answer to every situation. The models we use in therapy are informed by understandings of the broader social and environmental contexts in which people live; we conduct therapy with people when we believe that this will lead to improvements in their situation. However, we are also hopefully aware of the ways in which the broader context causes and maintains distress and impairment, and our therapies are often just an individual-level solution to a much broader problem. Therefore, working within the field of CBT can also mean looking beyond the narrow focus of the therapy itself to the impacts of the broader environments on mental health and leading fulfilling lives, including advocacy, political lobbying, and activism towards creating contexts in which the need for CBT is reduced. The past year or so has seen many such issues brought to the fore of international attention, including climate change, systemic racism, violence towards minority groups, and exacerbation of the increasing inequalities both globally and within countries by the pandemic. A challenge is to keep working on improving CBT as therapy, but also to be working to create conditions in which such therapy is rarely needed. Standing at this crossroads, we can ask ourselves whether we are content to restrict ourselves to selecting the route that feels safest to us (e.g., considering only therapy and therapy outcomes), or take “the one less traveled” (to quote Robert Frost), perhaps even building new routes (or, given the role of climate change, cycle paths) into territory that is less familiar, but allows us to engage more fully with the broader context and world through which we travel?

## Concluding Remarks

We hope that this introduction helps to set the scene for the collection of papers that make up this special issue “CBT at the Crossroads,” and itself stimulates reflection on and discussion about the field of CBT. We look forward to meeting you at future crossroads—whether at CBT conferences or elsewhere—to continue these exchanges, and in doing so help shape the routes ahead.

## Footnotes

1 We note that the terms “conference” and “congress” are largely interchangeable, with “congress” having perhaps wider international usage—as reflected in WCBCT’s title. In this article we use conference as a more general term, and congress when referring to WCBCT specifically.

2 We note that the next world congress, to be held at Jeju Island, South Korea, in 2022, in fact switches the “B” and “C” (and drops a “u’”) to become the 10^th^ World Congress of Cognitive and Behavioral Therapies (http://www.wccbt2022.org/). Whether this reflects an overtaking of “behavioral” by “cognitive” in terms of perceived importance, the naming convention of the Asian (and in fact Australian) CBT umbrella associations, or something else entirely, we will leave to the reader to consider. In any case, the use of “the” and “Therapies” highlighted here is retained.
